# Applying a Modern Pedagogy to Emergency Residency Ultrasound Education

**DOI:** 10.7759/cureus.22758

**Published:** 2022-03-01

**Authors:** Steven E Johnson, Dorthy Lakis, Jessica Kuxhause, Amit Bahl

**Affiliations:** 1 Emergency Medicine, Beaumont Health, Royal Oak, USA

**Keywords:** audience response, resident education, ultrasound, emergency, flipped-classroom

## Abstract

Background

Modern technology has revolutionized pedagogy in medicine. With the availability of high-quality resources in the palm of our hands, the flipped classroom model has gained widespread support. Additionally, devices such as “clickers” allow for the ability to interact much more directly with lecture participants.

Objective

We aimed to investigate the impact of a modern refresh to our emergency point of care ultrasound (POCUS) curriculum on resident exam performance and satisfaction.

Methods

In 2021, we conducted a single-site pre-post interventional study with Emergency residents as eligible participants. The interventions included two modifications to the POCUS curriculum. First, residents prepared and delivered mini-lectures in lieu of formal didactic sessions. Second, weekly image review was reimplemented with more active participation. Our primary outcome was end-of-rotation exam performance and our secondary outcome was learner satisfaction.

Results

During the study period, 19 residents participated in the curriculum. Exam scores were similar, 90.84% +/- 2.27% and 89.34% +/- 3.43% (p = 0.105) for pre and post-intervention scores respectively. Satisfaction surveys were completed by 12 of the 19 participants. On a scale of 1-5, average satisfaction was fair (3.9 to 4.1) regarding the mini-lecture intervention and high (4.3 to 4.6) for the active weekly image review modification.

Conclusions

The POCUS curriculum refresh was well-received by emergency medicine residents. However, there was no clear competency benefit when compared to the traditional approach. The modified active weekly review was particularly well-received amongst our residents and maybe a strong model for emergency POCUS programs across the country.

## Introduction

While pedagogy in medicine has seen dramatic shifts over the past decade, graduate medical education has been slow to integrate many of these updated teaching methods and models. Perhaps one of the most widely discussed modern teaching methods in medical education is the flipped classroom model [1-5]. In this model, the learner first prepares and reviews selected materials, then applies this knowledge to new problems during a planned educational session [5]. This was investigated in an internal medicine setting by Blair et al. in 2019, demonstrating improved scores after implementing a flipped classroom model in the education of antidiabetic prescription practices. In a meta-analysis evaluating flipped classroom models and student outcomes, it was shown that the flipped classroom method of teaching resulted in higher student satisfaction and higher pass rates [6]. Additionally, modern technology such as “clickers” and interactive learning games have revolutionized the way that teachers are able to interact with learners [7]. Specifically, an audience response system (ARS) is one way to engage learners in image and video interpretation. ARS technology has improved through the decades from portable response “clickers” to now incorporating smartphone and web applications [8]. Benefits ascribed to this strategy include increased participation, anonymity, measurable performance, and real-time feedback [7].

There may be a role to apply these innovative strategies to point of care ultrasound (POCUS) education. POCUS is a newer discipline in medicine with expansive growth over the past decade resulting in a plethora of high-quality, free, online resources readily available to learners. Thus, ultrasound education is particularly well suited for using the flipped classroom model. POCUS education traditionally involves bedside scanning, observations, and discussion of findings as well as formal didactic sessions. Beyond classic didactic sessions, organized ultrasound video review is a well-accepted method of teaching emergency residents about POCUS [4]. However, there is minimal discussion in the literature on how to approach this type of teaching. Classically, “tape” review sessions may consist of ultrasound faculty reviewing and discussing the POCUS images obtained during the week with the learners. Learner engagement during these reviews can be inconsistent, dependent on many factors including teacher/learner experience levels, interest, and timing. Additionally, depending on the scope of pathology versus “normal” images, redundancy of scan types and “normal” exams may have diminished teaching value,

We aim to determine if modern refresh utilizing technology and a flipped classroom model would result in better test scores and to determine if these interventions improved learner satisfaction on emergency medicine (EM) POCUS rotation.

## Materials and methods

Study design, setting, and participants

This prospective observational pre-post study investigated the impact of novel teaching methods on end-of rotation examination scores and learner satisfaction. The study was conducted at an academic tertiary care center with approximately 130,000 emergency department visits annually.

Emergency medicine residents who participated in the emergency ultrasound rotation during the interventional study period were eligible study participants. Residents participated on a voluntary basis. Any participant who did not wish to participate in the research study was able to exclude themselves from data collection without any negative effect on their evaluation for the rotation and could choose not to respond to the optional anonymous survey. The study was approved by Beaumont Health’s Institutional Review Board (approval number 2021-002).

The Beaumont Royal Oak Emergency Medicine Residency Program requires two, two-week-long dedicated ultrasound rotations across a resident’s postgraduate year 1 (PGY1) and PGY2 years. There are 14 residents per PGY class. During each two-week rotation, there are generally two-three residents. Of all 28 potential participants, 19 residents attended the rotation after the intervention was implemented. The objective of the rotation is to perform educational ultrasound scans in the emergency department and gain fundamental knowledge regarding POCUS as an emergency physician. At rotation end, all residents complete a multiple-choice digital exam to assess their knowledge. Aligned with American College of Emergency Physicians (ACEP) guidelines, we require all residents to log >150 POCUS studies during their three years of residency. During their senior year, residents frequently choose to do an additional 2 weeks of ultrasound as an elective, and most residents perform between 300-500 POCUS studies prior to graduation based on historical numbers from our resident ultrasound logs.

Our program has 13 POCUS machines available for resident use as well as all major types of ultrasound transducers, including an endocavitary and transesophageal echo probe. We have a digital image archiving solution, Qpath-E (Telexy Healthcare Inc., Coquitlam, Canada), that allows for the inclusion of POCUS images in the patient’s electronic medical record as well as archival of educational studies for teaching. 

Intervention

From January through June of 2021, we implemented a modern refresh to the emergency ultrasound POCUS educational curriculum including two main changes to the traditional curriculum. First, we instituted a flipped classroom model in which residents were responsible for delivering daily “mini-lectures” to each other. Second, the weekly “tape” review session was modified to improve audience interaction via a modern learning game (Aha-Slides) and a process of hand-selecting scans for review [9]. 

Prior to our intervention, didactics in this rotation were typical lecture-style using the “expert to novice” approach. Each morning the available ultrasound faculty would give a 10-15 minute lecture on a topic of their choice prior to scanning for the day. For our flipped classroom intervention, formal daily didactics were replaced with “mini-lectures” from each resident. Mini-lectures were developed by residents based on any educational materials they were able to find. During their two-week rotation, each resident was assigned a simple POCUS topic each day. The goal was for the resident to review the topic independently each evening, then provide a five-minute lecture on the topic the following morning. Topics were customized based on PGY level as well as individual resident interests. These “mini-lectures” were done interactively, with the residents utilizing a whiteboard and engaging the audience. These sessions were attended by all rotation participants as well as any ultrasound faculty available that day.

The process of weekly video review was modified in two major ways in an effort to improve resident engagement. Our previous approach consisted of reviewing all scans performed during the prior week as a group led by an ultrasound faculty member. This approach typically involved a review of numerous back-to-back “normal” scans and had no pre-planned or focused topics from faculty members. We felt that the monotony and lack of focused educational content of this format led to inconsistent learner participation and a general lack of enthusiasm. In our modified curriculum, first, prior to the weekly session, the ultrasound faculty leader would review all potential videos for review. The goal of this preparation session was to eliminate redundant “normal” images and allow the faculty to design a cohesive image review session that covered all relevant topics in a timely manner. Second, using a web-based ARS (Aha-slides), all residents independently and actively answered ultrasound questions throughout the review session. At the beginning of the session, the ultrasound faculty leader would initiate an ARS session and instruct all participants to log in. Participants included rotating residents, fellows, and any additional faculty that were present for review. Throughout the session, the ultrasound faculty leader would pose questions to the participants based on the ultrasound video clip being reviewed. Participants could then submit their individual answers to the ARS and after all the submissions were received these answers were reviewed as a group.

Data collection

We collected end-of-rotation written examination scores for each study participant from the year prior to the intervention. The written examination consisted of 100 multiple choice and free-text-style questions covering a broad range of core and advanced ultrasound applications. We then prospectively collected examination scores from the same exam questions post-implementation of the new teaching methods. 

**Figure 1:**
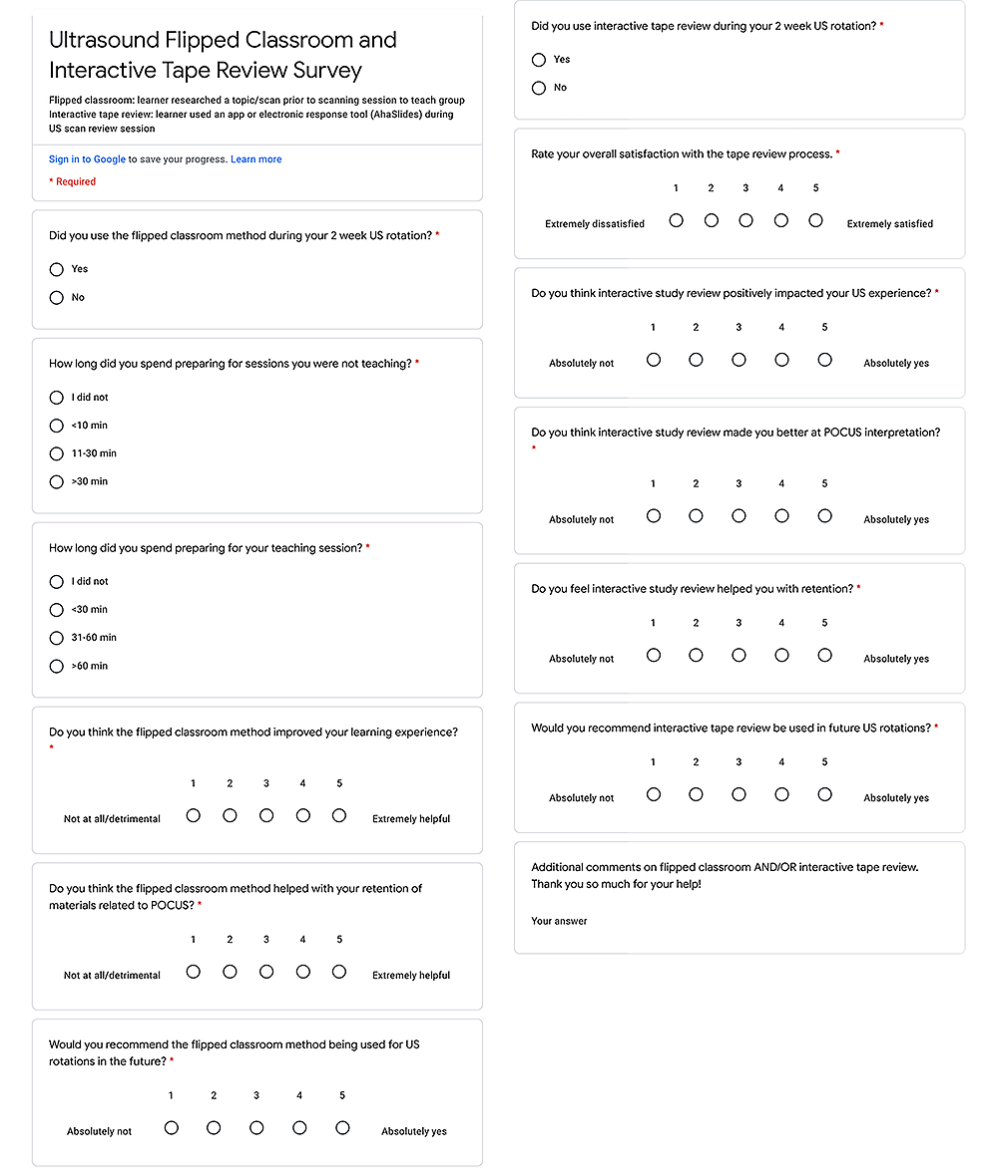
Resident ultrasound education survey Google Forms survey regarding resident flipped classroom and interactive tape review experience.

We also gathered anonymous survey data from participants after undergoing the POCUS refresh curriculum (Figure 1). The survey was created in Google Forms and was emailed to all participants at the conclusion of the study period. Questions regarding the flipped classroom teaching sessions focused on time spent preparing the materials and learner satisfaction with the process. The responses to these questions were pre-determined and categorized based on the amount of time spent preparing for each session (<30min, 31-60min, >60min). Responses to questions related to satisfaction with the program were categorized on a 1 to 5 scale.

Outcomes

The primary outcome was end-of-rotation examination performance. Exam score was reported as the percentage of correct answers divided by the total number of questions. The secondary outcome was resident satisfaction with the new teaching methodology. Satisfaction was categorized on a numerical 5-point scale with 5 being most satisfied and 1 being least satisfied. 

Statistical analysis

The primary outcome was evaluated by comparing pre-and post-intervention exam scores between PGY years, utilizing a t-test analysis. Mean scores, represented as a percentage of correct answers, were compared between the pre-and post-intervention groups. Microsoft Excel was utilized for statistical analysis. After the degrees of freedom and the t-value were determined, a p-value was found using a table of values from the Student’s t-distribution. A predetermined p-value of ≤0.05 was chosen to represent statistical significance. A value >0.05 would represent a lack of statistical significance and acceptance of the null hypothesis.

Regarding the secondary outcome, survey responses were represented as averages or percentages where appropriate. Survey questions regarding preparation time were categorical (none, <30min, 31-60 min, and >60 min) and responses were aggregated as a percentage. Survey questions regarding learner satisfaction were scaled from categorical (1 for worst to 5 for best) and summarized as an average value.

## Results

During the one year prior to the intervention, there were 27 total ultrasound exam scores available. These scores included those of 13 PGY1 EM residents and 14 PGY2 EM residents. During the study period, scores were collected for a total of 19 participants. These included 10 PGY1 EM residents and 9 PGY2 EM residents. Overall average score was 90.84% +/- 2.27% and 89.34% +/- 3.43% (p = 0.105) pre-and post-intervention respectively (Table 1).

**Table 1:** End of Rotation Exam Scores Comparison of end or rotation exam scores pre and post-implementation of the new POCUS curriculum. PGY 1: Postgraduate year 1; PGY 2: Postgraduate year 2

	Overall Average with SD (as % score)	PGY 1	PGY 2
Pre-implementation	90.84% +/- 2.27%	90.33% +/- 2.49%	91.31% +/- 2.00%
Post-implementation	89.34% +/- 3.43%	88.49% +/- 3.58%	90.27% +/- 3.18%
p-value	0.105	0.186	0.398

**Figure 2:**
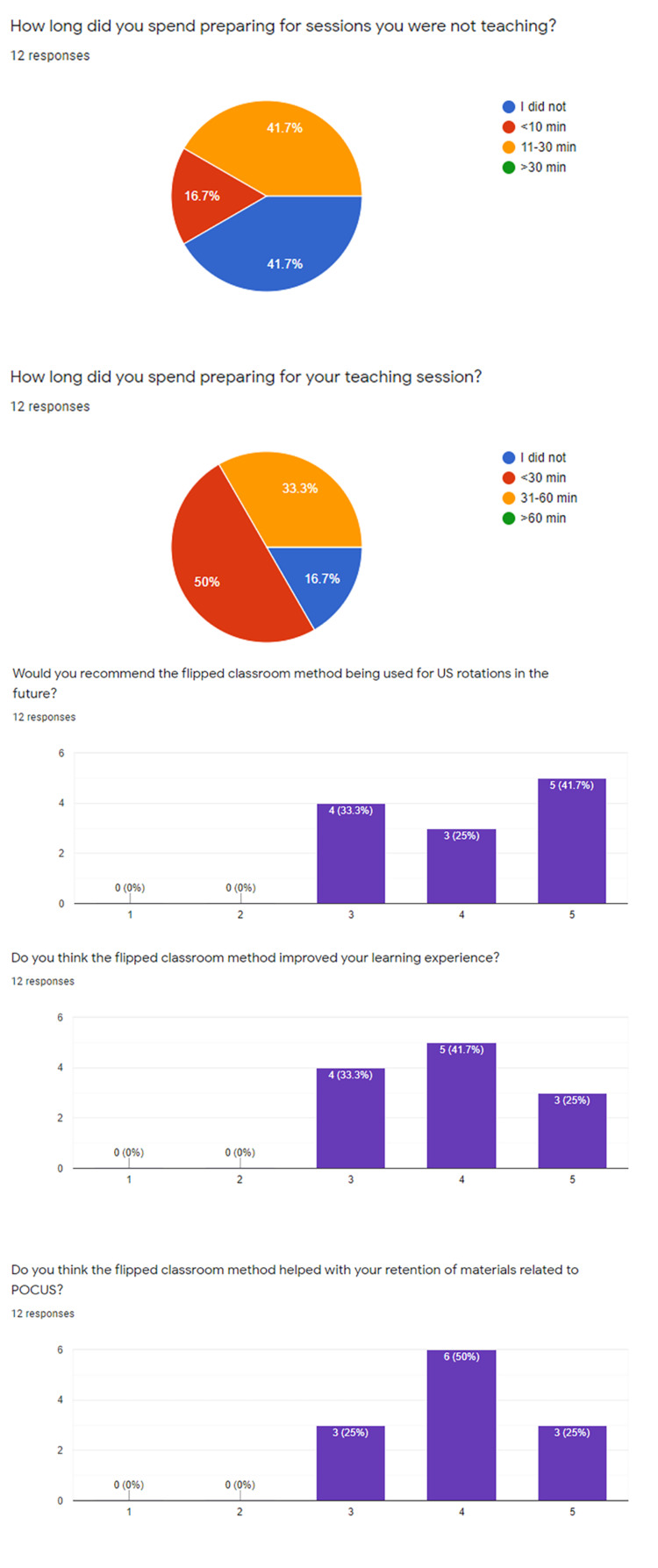
Survey results regarding the flipped classroom experience Breakdown of survey responses from residents regarding the flipped classroom experience. US: Ultrasound; POCUS: Point of care ultrasound

**Figure 3:**
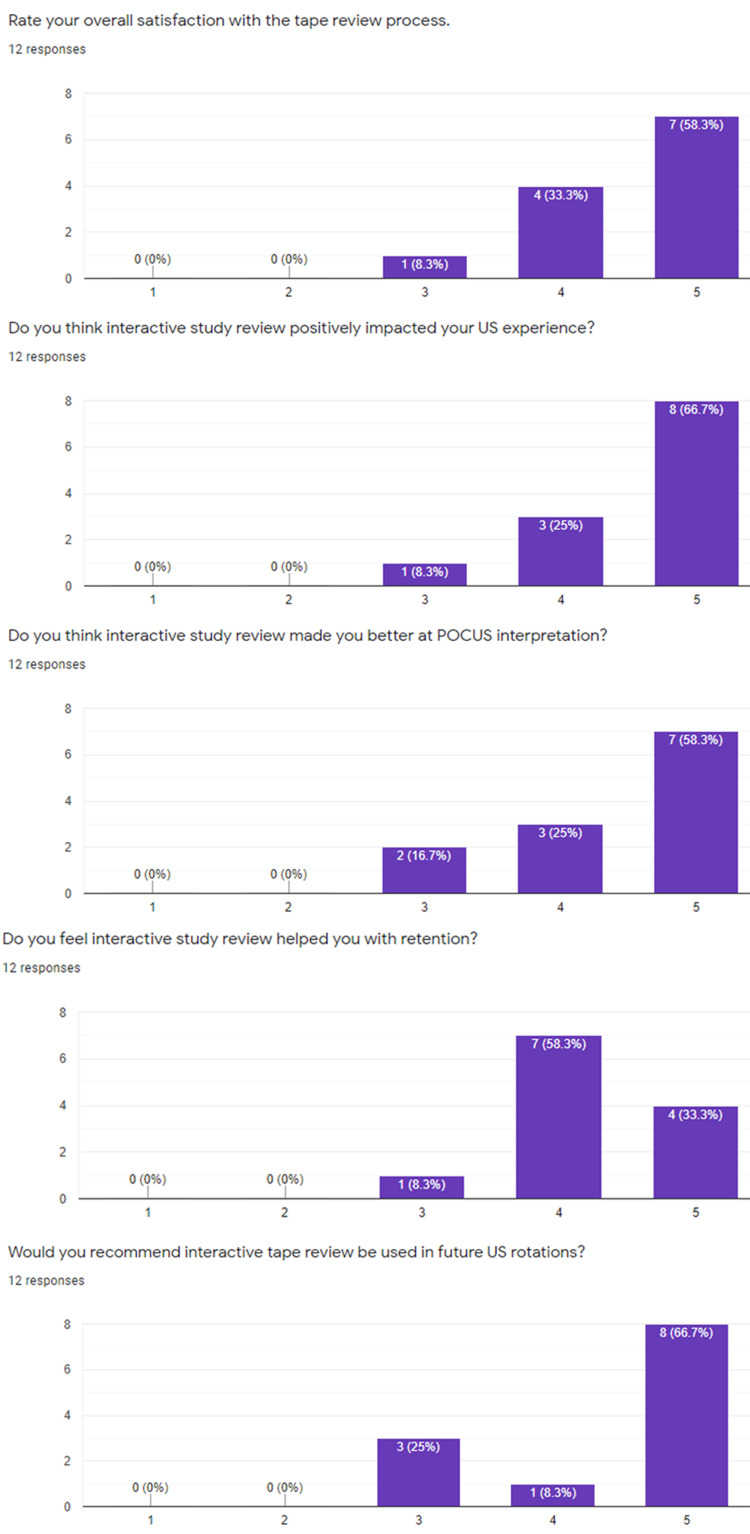
Survey results regarding the modified tape review process Breakdown of survey responses from the residents regarding the modified tape review process. US: Ultrasound; POCUS: Point of care ultrasound

Surveys were completed by 12 of 19 participants and are displayed in Figures 2, 3. The average time spent preparing for the flipped classroom sessions was 20 minutes amongst all respondents. Regarding the question “Do you think the flipped classroom method improved your learning experience?” The average rating was 3.9, with four respondents indicating a 3, five answering 4, and 3 of the responses indicating 5, or “Extremely helpful.” The question, “would you recommend the flipped classroom method…” received slightly higher marks with an average score of 4.1 and five respondents indicating 5, or “Absolutely yes” to this question. Overall satisfaction scores with the modified “tape” review were higher, averaging >4 for all questions. Specifically, the question, “Do you think interactive review positively impacted your ultrasound experience?” received an average score of 4.6 with the 8 of 12 respondents indicating a score of 5, or “Absolutely yes.”

## Discussion

Our results demonstrate that the flipped classroom model is an effective method of teaching emergency medicine residents POCUS concepts. While we did not see a statistically significant increase in test scores among our cohort after implementation of the program, given our small number of participants (19), it is possible that with an increased sample size this may have changed. Regardless, the lack of statistical difference between pre and post-implementation scores demonstrates that the flipped classroom method is at least as effective as previous educational methods and with an average recommendation score of 4.1, this method appears very enjoyable to the learner. Unfortunately, we do not have survey data for the previous style of image review to compare.

Previous literature has suggested that the learners may dislike the flipped classroom model due to the amount of time required outside of the classroom [3,10]. Our survey results showed that even with the limited request of 5-10 minute “mini-lectures,” learners required a moderate amount of time outside the classroom to prepare these. Most residents spent approximately one hour each evening preparing for their own sessions and reviewing materials relevant to their colleagues’ sessions. However, overall, it appears that despite the time required outside of the classroom, residents responded favorably when asked if the flipped classroom method improved their learning experience with an average response score of 4.

Perhaps the more meaningful outcome identified in this study was the overwhelmingly positive response to the modified “tape” review curriculum. We found that the majority of respondents felt that the modified video review sessions positively impacted their overall ultrasound rotation experience. To our knowledge, there is no existing literature or descriptions of ultrasound video review sessions, despite the fact that they are a ubiquitous method of teaching ultrasound curriculum [4]. Our refreshed approach to “tape” review incorporates modern pedagogy along with education technology to maximize the potential of these sessions. While ARS technology has become a popular tool during lectures, to our knowledge there is no literature describing its use in this fashion. Additionally, survey responses indicated that residents felt these sessions improved their ability to interpret ultrasound images.

Our study had some limitations. This was a small sample size cohort. It is possible that with a larger sample size there would have been more of an impact on outcomes, either positive or negative. This was a single-institution study, therefore potentially limiting its applicability to other educators at other institutions. Additionally, we did not validate or assess our survey prior to its implementation. Finally, the implementation of simultaneous changes to the curriculum may have introduced some additional confounders that may have skewed our survey results.

## Conclusions

Overall, the POCUS curriculum refresh was well-received by emergency medicine residents. While there was no statistically significant benefit to exam score post intervention, our small sample size may have limited our ability to detect a meaningful impact. The modified “tape” review sessions were particularly well received amongst our residents. We hope that our experiences and curriculum can serve as a model for emergency POCUS programs across the country.
